# A Process-Based Approach to Predicting the Effect of Climate Change on the Distribution of an Invasive Allergenic Plant in Europe

**DOI:** 10.1371/journal.pone.0088156

**Published:** 2014-02-12

**Authors:** Jonathan Storkey, Pierre Stratonovitch, Daniel S. Chapman, Francesco Vidotto, Mikhail A. Semenov

**Affiliations:** 1 AgroEcology Department, Rothamsted Research, Harpenden, Hertfordshire, United Kingdom; 2 Computational and Systems Biology Department, Rothamsted Research, Harpenden, Hertfordshire, United Kingdom; 3 Centre for Ecology and Hydrology, Edinburgh, United Kingdom; 4 University of Turin, Grugliasco, Italy; Cirad, France

## Abstract

*Ambrosia artemisiifolia* is an invasive weed in Europe with highly allergenic pollen. Populations are currently well established and cause significant health problems in the French Rhône valley, Austria, Hungary and Croatia but transient or casual introduced populations are also found in more Northern and Eastern European countries. A process-based model of weed growth, competition and population dynamics was used to predict the future potential for range expansion of *A.artemisiifolia* under climate change scenarios. The model predicted a northward shift in the available climatic niche for populations to establish and persist, creating a risk of increased health problems in countries including the UK and Denmark. This was accompanied by an increase in relative pollen production at the northern edge of its range. The southern European limit for *A.artemisiifolia* was not expected to change; populations continued to be limited by drought stress in Spain and Southern Italy. The process-based approach to modelling the impact of climate change on plant populations has the advantage over correlative species distribution models of being able to capture interactions of climate, land use and plant competition at the local scale. However, for this potential to be fully realised, additional empirical data are required on competitive dynamics of *A.artemisiifolia* in different crops and ruderal plant communities and its capacity to adapt to local conditions.

## Introduction

Climate change may impact the severity of pollen induced atopic disease by affecting the large scale distribution and local prevalence of allergenic species, the timing and amount of pollen produced and the allergenicity of individual pollen grains. A species of particular concern in Europe is *Ambrosia artemisiifolia* L. (common ragweed), an alien plant in Europe that has expanded its range over recent decades. It is now responsible for significant health and economic impacts in the most infected areas namely a) in the Pannonian Plain in Central Europe including Hungary and neighbouring countries especially Serbia, Croatia, Slovenia, Slovakia and Romania [1], [2], [3], [4], b) in the Rhône Valley in France [5] and c) in Western Lombardy, Italy [6]. *A.artemisiifolia* is an annual plant with origins in North America. Although it was first observed in Europe in the mid 19^th^ Century, it began to spread rapidly in Europe after 1940 via transportation networks and contaminated crop seed [7]. It is highly invasive with allergenic pollen that causes hay fever, asthma and atopic dermatitis [8]. Once established in a country, control measures are labour intensive and expensive [9] and there are benefits to anticipating the potential future distribution and impact of the species under climate change to inform surveillance of regions that are vulnerable to populations establishing.

The conventional approach to predicting changes in plant distribution under climate change has been to develop species distribution models (SDMs) based on the current range of a species characterised by their habitat requirements. Climate change scenarios can then be applied to these models to predict the change in the bioclimatic envelope and the resulting shift in community composition [10]. A number of habitat models have been developed for *A.artemisiifolia* that also incorporate dispersion dynamics [11], [12], these models are valuable for predicting future distribution based on physiological thresholds and rate of spread. A more mechanistic phenological approach was also recently developed by Chapman *et al.* [13] who predicted the future distribution of *A.artemisiifolia* based on the likelihood of different stages of the life cycle being completed. However, as well as being constrained by physiological tolerance thresholds, the probability of a species establishing in a locality will be determined by other factors such local soil properties and management and biotic interactions including competition with crops and the native flora [14], [15]. These additional drivers of community assembly effectively mean that the realised niche for a species may be considerably smaller than its fundamental niche determined by physiological tolerances. Local environment and management factors are particularly relevant for modelling the fitness of populations of a ruderal species such as *A.artemisiifolia* that is adapted to managed habitats such as cropped fields and can be rapidly out competed by native species [16]. Therefore, while SDMs are a useful first approximation, ideally, process-based models that can operate on a smaller scale are required to predict the effects of local resource heterogeneity, land management and biotic interactions on plant community assembly [17]. In the context of predicting the future impact of allergenic plant species, process-based models also have the advantage of quantifying the response of plant growth and, therefore, pollen production to changes in climate or management.

A process-based model of plant growth, competition and population dynamics, Sirius 2010, has previously been developed to predict the impact of climate change on the damage niche of an agricultural weed, *Alopecurus myosuroides* (Huds.) [18]. The model predictions highlighted the importance of considering local environmental conditions as the response of the weed to climate change was confounded by variation in soil properties. Sirius 2010 has a modular structure and the algorithms which describe plant growth and development are generic. Assuming sufficient empirical data are available to parameterise and calibrate the model, therefore, it can be used to simulate the response of any annual plant species to climatic and environmental variation. In this paper, we present an adaptation of Sirius 2010 for *A. artemisiifolia*, growing in monoculture stands, and predict the potential for range expansion under climate change based on the population growth rate (λ) calculated across Europe. Although, in reality, *A.artemisiifolia* will be found growing in plant communities (either alongside transport routes or in crops), our aim in this first iteration of the model was to quantify the potential niche given the most favourable growing conditions. The model is also used to predict the relative change in pollen production under climate change scenarios.

## Materials and Methods

### Modelling *A.artemisiifolia* growth, development and population dynamics

Sirius 2010 is a plant growth simulation model capable of modelling inter-plant competition for light, water and nutrients based on a wheat growth model [19], [20] and using functions for competition for resources from the INTERCOM model of crop-weed competition [21], [22]. A mechanistic approach is taken to modelling the transition between life stages which captures the interactions of weather and management on biological processes. Plant growth and development is simulated on a daily time step and plants described as a collection of organs (roots, leaves, stems, flowers and seeds). The model has the capability to simulate competition for resources between multiple species in the canopy; for the initial model runs presented here, however, *A.artemisiifolia* is assumed to be growing in a single species stand. The growth of individual organs is predicted from resource demand, calculated as a function of developmental stage and potential growth rate under unlimiting conditions, and resource supply. The equations describing resource capture and the impact of limiting factors on resource use efficiency (CO_2_, temperature and water) are described in detail in Stratonovitch *et al.* [18]. Here, we focus on the modifications made to the model to simulate specific aspects of the biology of *A.artemisiifolia* and sources of data for parameterisation.

*A.artemisiifolia* is a frost sensitive, summer annual that is adapted to avoid emergence in the autumn. This is achieved by seeds having primary dormancy at the time of shedding and requiring a period of chilling to break the dormant state. The optimum temperature for breaking dormancy has been determined experimentally as 4°C; slower dormancy release was observed at temperatures above 5°C or below 0°C [23]. In the model, a period of 12 weeks is required at this temperature before seeds are able to germinate. Sub-optimal temperatures are also effective but take longer. A function describing the rate of dormancy release (d^−1^) at different temperatures based on the results of Willemsen [23] was included in Sirius 2010. Once the threshold for the breaking of dormancy has been reached, the rate of germination was modelled as a Weibull function using hydrothermal time that integrates the effect of temperature and soil moisture (Equation 1):
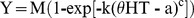
(1)Where *Y* is cumulative germination (%) at a hydrothermal time (*θ*_HT_), *M* is maximum germination, *k* is rate of increase, *a* is lag phase and *c* is shape parameter.

The calculation of *θ*_HT_ requires parameters for the base temperature and moisture content below which *θ*_HT_ does not accumulate. These values have been determined experimentally as 3.6°C and −0.8 mPa respectively [24]. The proportion of seeds emerging from the seed-bank was modelled as a stochastic function with a left skewed distribution and a median of 26%. This was calculated as a generic distribution using data from multiple weed species across several sites and years.

Phenological development of *A.artemisiifolia* is primarily driven by temperature [25] with estimated base and optimum temperatures of 0.9°C and 31.7°C [26]. Previous authors have also found a relationship with photoperiod; *A.artemisiifolia* is characterised as a short daylength plant and flowering is predicted to be delayed when daylength exceeds 14.5 hours [13]. These functions predict a positive correlation between the onset of flowering, measured in Julian days, and latitude. However, data from the US on *A.artemisiifolia* flowering times [27] and from Europe on pollen production do not support this prediction and imply that regional biotypes are adapted to local conditions, synchronising flowering time. Our model, therefore, used a standard calendar date for the onset of flowering (1^st^ of August). *A.artemisiifolia* is frost sensitive and the end of flowering has been found to be correlated spatially and temporally with the onset of the first frost [27]. We imposed a number of rules that simulated the end of flowering and plant senescence based on physiological thresholds and resource availability: 1) threshold of accumulated thermal time reached; 2) low temperature thresholds, i.e. daily minimum temperature is below 0°C, or 5-day mean minimum temperature is below 7°C; 3) severe drought, i.e. 10-day mean of the ratio between actual and potential transpiration is below 0.1. These rules terminate the flowering season and therefore stop pollen and seed production. They have been parameterized to reproduce observed end of pollen seasons for a range of latitudes in North America and Europe. From germination, the plant is killed by frost (daily min air temperature ≤0°C) and drought when it transpires less than 10% of the potential transpiration over 10 days. The relationship between thermal time and transition between developmental stages, with the associated shift in allocation of resources between plant organs, was based on empirical data from field experiments in Michigan, US [28]. *A.artemisiifolia* is a monoecious plant (having the female and male reproductive organs separated in different floral structures on the same plant). The relationship between plant mature biomass and pollen and seed production is allometric and has been quantified from empirical data [29]. Losses of fresh seed from predation were modelled as a stochastic function with a normal distribution (mean 0.9 and standard deviation 0.1) and decline of the old seedbank as an exponential function with a half-life of 17 years [30]. The model was used to calculate the fitness of a local population using a measure of the population growth rate, λ, calculated as seed number at time *t_+_*_1_/seed number at time *t*_0_.

The data for initial parameterisation of the model were largely derived from studies in the US. It was important, therefore, to calibrate the model for European populations of *A.artemisiifolia*. Growth analysis data from two field experiments in 2006 and 2007 at Grugliasco (Turin, Italy: 45°03′53″N, 7°35′38″E) sampling monoculture stands of *A.artemisiifolia* were, therefore, used in the final model development. The model was fitted to observed data for emergence date, flowering time, height and biomass allocation to different plant organs.

### Local scale climate scenarios and modelling European distribution

To model the European distribution of *A. artemisiifolia*, Sirius 2010 was used to evaluate the population growth rate (λ) as a function of seed germination rates, production of new seed and seed losses driven by local climate variables. From the ELPIS dataset [31], 479 locations were randomly selected with a minimum neighbouring distance of 100 km. For each location, 100 years of daily synthetic weather were generated using LARS-WG [32]. The asymptotic population growth rate (Λ) was calculated as the mean of log(λ) over 1000 yearly simulations, each using a random combination of weather data generated for one growing season and one germination and seed loss rate. Two additional statistics were also computed: end of pollen season and seasonal pollen production. From the 479 random locations, a subset of 25 sites across Europe was used to categorise Λ values into a suitability index: U.0 – highly unsuitable, U.1 – unsuitable, C.0 – casual (less likely), C.1 – casual, E.0 – established, E.1 – well established. Ranges for these categories were derived by comparing generated Λ in baseline climatic conditions at these 25 sites to the level of abundance of *A.artemisiifolia* from the map of current distribution.

## Results

**Figure 1:**
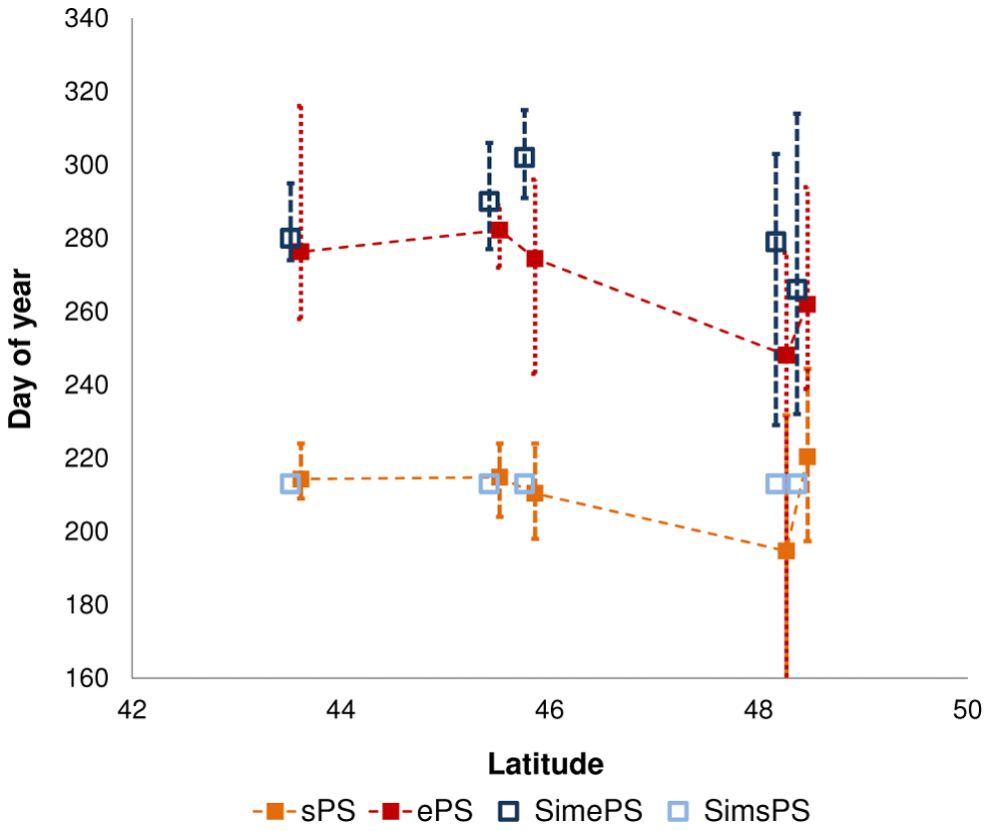
Mean values for observed start (sPS) and end (ePS) of pollen season of *A.artemisiifolia*, from up to 20 years of data, along a latitudinal gradient and start and end simulated by the process-based model (SimsPS, SimePS). Error bars represent maximum and minimum observed and simulated values.

The observed data of the start of the *A.artemisiifolia* pollen season only covered a relatively small latitudinal gradient and the correlation between latitude and the onset of flowering expected from a previous phenological model was not observed (Figure 1). Although it would be instructive to perform a similar analysis with data on flowering times across a wider latitudinal gradient in Europe, our results agree with data on *Ambrosia spp.* flowering times from the US where the onset of flowering could largely be predicted on the basis of calendar day suggesting local populations may have adapted to synchronise development [27]. The observed data from European pollen monitoring stations had high year to year variability but predicted values for the end of pollen production, using the three rules, generally fell within the range of observed values (Figure 1).

**Figure 2:**
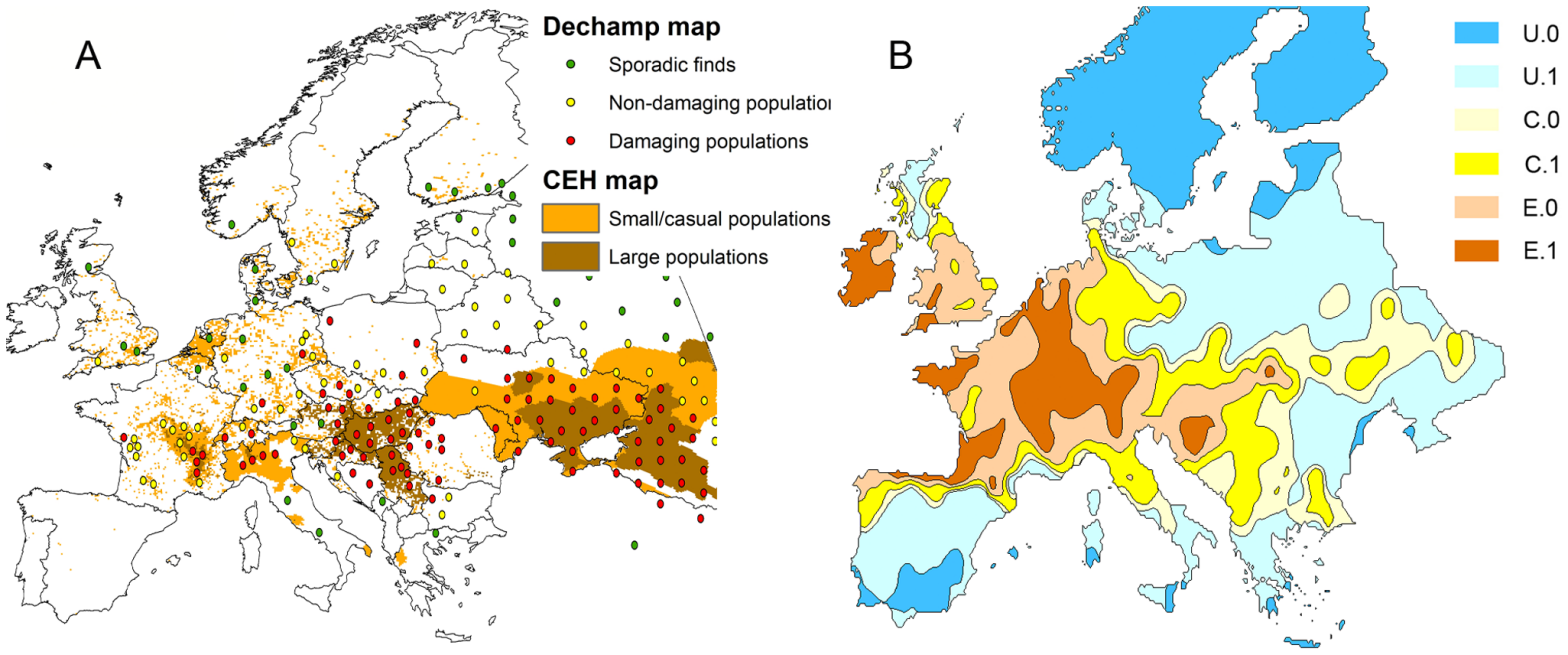
Potential climatic niche of *Ambrosia artemisiifolia* under baseline conditions compared to observed distribution. A. Combination of two recent maps showing the approximate distribution of *A.artemisiifolia* in Europe compiled by Déchamp [44] and Bullock et al at the Centre for Ecology and Hydrology (CEH) [35]. The quality of observed data used to generate the maps varies with good quality data obtained from northern, central and western Europe and poorer quality data from Russia and Ukraine. B. Predicted potential range of *A.artemisiifolia* from output of process based model assuming that appropriate habitat in terms of land use is available at all sites. Actual distribution will be modified by cropping patterns and level of control. The categories are: U.0 - highly unsuitable, U.1 - unsuitable, C.0 - casual (less likely), C.1 - casual, E.0 - established, E.1 - well established.

The predicted distribution defined using the six categories of suitability from ‘unsuitable’ to ‘well established’ using baseline climatic data corresponded reasonably well with the latest observed distribution maps (Figure 2). Centres of high likelihood of occurrence were identified as the Rhône valley in France, an area in and around Hungary and Switzerland and the Netherlands. Although giving an indication of the validity of the model output, Figures 1a and 1b cannot be directly compared statistically, because they represent different quantities. Figure 1a shows observed grids or sites where ambrosia has been detected and, for the majority of grids/sites, there is no information provided on whether the population is established or casual. In addition, because the invasion event is on-going, the observed distribution is expected to change over coming decades whereas Figure 2b predicts the potential distribution once the invasion is complete. It is also likely that the distribution map in Figure 1a is biased by sampling effort; for example, it is likely that populations in the South-eastern corner of the maps in Ukraine may have been underrepresented [6].

Here we assume that there are no barriers to dispersal and all areas have suitable land use (ruderal habitats, especially spring sown annual crops) for *A.artemisiifolia* to persist. For some regions lacking suitable habitat the map of potential distribution predicts established populations of *A.artemisiifolia* where it has not been observed; for example, the south west of England and Southern Ireland are dominated by permanent grassland. Incorporating land use factors at a finer scale would further refine the predictions but will not affect the predicted northern, southern and eastern limits of its range. These correspond well with reported data on the extent of the range of *A.artemisiifolia* with isolated casual populations extending as far as south as northern Spain and Italy and north up to the UK and Sweden [33], [34]. The model predicted the southern limit to be driven by moisture stress and the northern limit by insufficient thermal time for seed to mature. Importantly, there were no areas where *A.artemisiifolia* is currently an established problem and where the model predicts unsustainable populations with the possible exception of Russia and Ukraine where data quality on observed populations used to generate the distribution map is poor [35].

**Figure 3:**
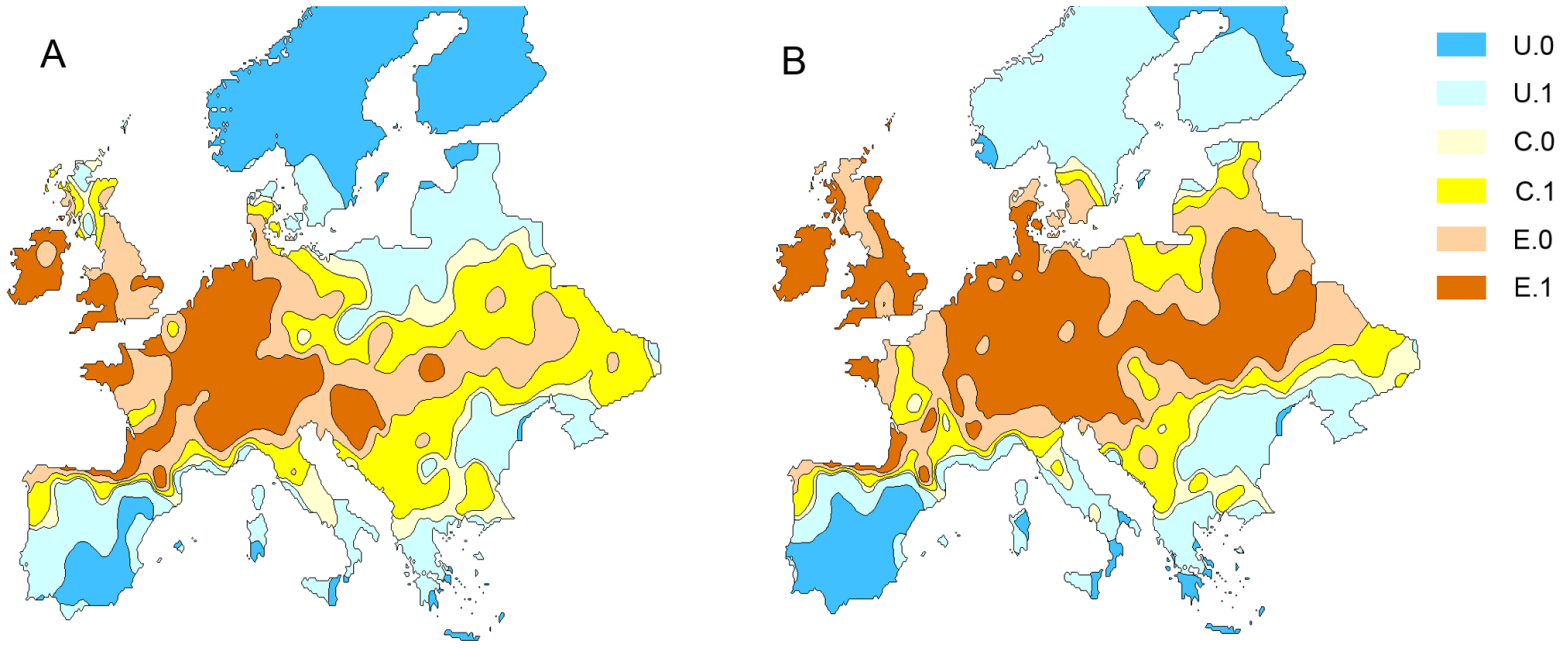
Distribution of *Ambrosia artemisiifolia* (common ragweed) in Europe under climate change as predicted by the process based model. A. Using HadCM3(A1B) scenarios for near future 2010–2030 and B. long-term future 2050–2070. The categories are: U.0 - highly unsuitable, U.1 - unsuitable, C.0 - casual (less likely), C.1 - casual, E.0 - established, E.1 - well established.

**Figure 4:**
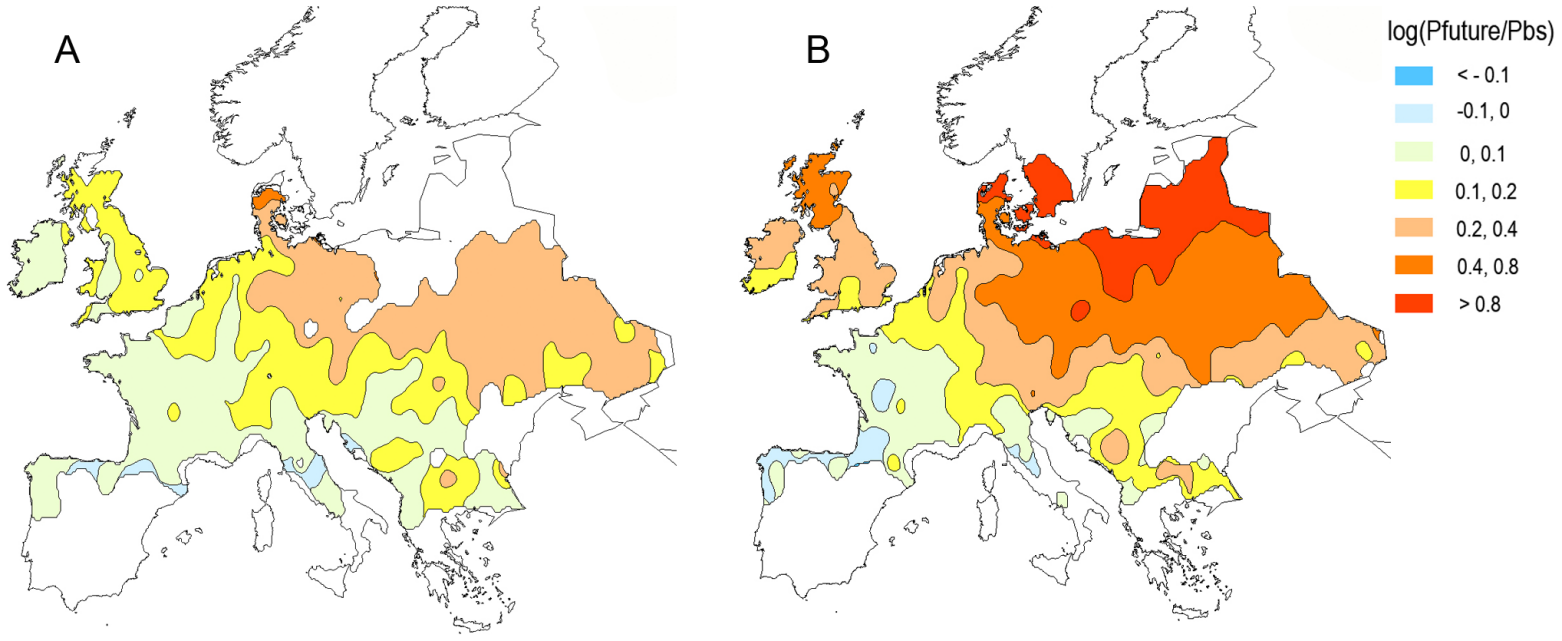
Logarithm of relative increase in *Ambrosia artemisiifolia* pollen production, Pfuture/Pbs, for the HadCM3 (A1B) scenarios. A. Near future (2010–2030) and B. long-term future (2050–2070), where Pbs and Pfuture are pollen production for the baseline and future climate scenarios. The model output is pollen production by the plant, there is no prediction of pollen emission or transportation included in the model. Only areas predicted to be suitable for *A.artemisiifolia* (casual or established) are shown. Where populations are predicted to colonise new areas, increased pollen production will therefore be indicated as >0.8 although absolute numbers of pollen grains may be relatively low when compared to well established populations.

Under the HadCM3(A1B) scenarios for 2010–2030 and 2050–2070, the southern limit of the range of *A.artemisiifolia* was predicted to change very little, as future rainfall patterns meant most of Spain, southern Italy and Greece remained too dry for *A.artemisiifolia* to maintain viable populations (Figure 3). However, the available suitable habitat for *A.artemisiifolia* was predicted to extend further north and east under climate change as increasing summer temperatures resulted in a faster accumulation of thermal time and a higher probability of the plant completing its life cycle and producing mature seed. Under these scenarios, it is likely that populations in Scandinavian countries and Britain that are currently casual may become established (Figure 3). Where *A.artemisiifolia* is currently well established and found at high densities, in Hungary, Croatia and the French Rhône Valley, the model predicted only a small increase in the capacity for increased pollen production under climate change (Figure 4). However, at the Northern extent of the range relatively large increases in pollen production were predicted potentially creating a health problem where *Ambrosia* pollen concentrations are currently below the threshold for inducing clinical symptoms.

## Discussion

The model output predicted a wider European distribution of *A.artemisiifolia* than is currently observed. Although, the actual distribution will be further modified by land use factors (see below), it is likely that the species has not yet filled the potential climatic space as the spread of *A.artemisiifolia* through Europe represents an on-going, dynamic invasion event [11]. Developing predictive models for the potential stable state of such a system under current conditions and future climate poses fundamental challenges for traditional correlative approaches. Specifically, models based on correlations between species occurrence and habitat parameters depend on the assumption that its current distribution is in equilibrium with its environment [36]. The alternative of using habitat data from the species' native range may also be problematic because of evidence of niche shifts between native and naturalised environments due to processes such as enemy release [37]. In contrast, forward process models, of the type developed here, based on first principles and parameterised from independent eco-physiological experiments have the potential to predict potential realised niches under novel environments with greater confidence as it can be assumed that the biological principles driving the model output are constant across environments [38]. However, these models fall at the extreme end of the tractability/complexity trade-off, with a high demand for data and parameterisation [39]. As a consequence, their development and application to species distribution modelling lags behind correlative models and they also often fall back on correlating missing parameters with the observed distribution [38].

Independently of the development of species distribution models in invasion ecology, detailed process based models of crop growth and development have been progressed over recent years to better understand the environmental and physiological constraints on crop yield [20]. These models have the power to capture the interaction of multiple management and environmental variables on a small temporal and spatial scale and they represent an important resource for predicting realised niches under future scenarios of invasive species. This potential has recently been demonstrated for an annual weed at a national scale [18]; here we have used the same approach to model the potential niche for a species currently invading a new continent. In so doing, we have addressed a number of challenges facing the application of forward models to invasion ecology discussed by Chapman *et al.* [13]. Specifically, the processes in the model are all parameterised using independent data sets and the use of local scale climate scenarios generated on a daily time step allows the impact of short term environmental stress on plant growth to be captured.

The simulation of the future distribution of *A.artemisiifolia* indicated a risk of established populations spreading to Northern European states where the species is currently only recorded as a casual alien, including the UK and Denmark, due to more favourable growing conditions and delayed frost. Whether this threat is realised will be a function of future land use and levels of control. *A.artemisiifolia* is a ‘ruderal’ species that relies on regular disturbance to persist [16]; land use is, therefore, an important driver of occurrence and it is commonly found in high densities along transport routes and as an arable weed in crops of sunflower (in the same family as *Ambrosia*, Compositae), maize and soybean. The competitive dynamics between plant species will be specific to these different situations and potentially affect the population growth rate and fitness of local populations of the weed. In addition, the level of control (either through physical removal or herbicides) will vary between region and country and have a large impact on the capacity of the weed to establish in a new location. For example, the herbicide load calculated as the total weight of active herbicide ingredients applied to cereals for Hungary (where *A.artemisiifolia* has become the most economically damaging arable weed) is 0.11 kg ha^−1^ compared to 0.78 for Germany and 1.34 for the United Kingdom [40]. Differences in the types of crops grown and level of weed control will not only mitigate the spread of *A.artemisiifolia* but also determine the regional health impacts through the amount of pollen produced. As such, the effects of climate change on the realised niche for *A.artemisiifolia* will be manifested as much by indirect effects on farmer behaviour as direct climatic effects on plant biology.

The process based approach to modelling the response of plant populations to climate change developed here has sufficient mechanistic detail to capture these interactions between local weather, management conditions and biotic interactions with neighbouring species. However, at present insufficient empirical data are available to calibrate the model for multispecies plant communities, including crop competition under future climate scenarios [41]. Future development of the model will allow the predictions of spread and impact (in terms of pollen emission and crop yield loss) to be further refined by incorporation of data on land use and crop management at the field scale. An additional advantage of our process based growth over conventional SDMs is the capacity to simulate biomass production and allocation, including the amount of pollen produced, based on published allometric relationships. This has enabled us to generate initial predictions of relative changes in the regional potential pollen production of *A.artemisiifolia* under climate change. However, the situation is complicated by the capacity of *A.artemisiifolia* to shift the allocation of resources between seed and pollen in response to the environment; *A.artemisiifolia* is a monoecious species (having the male and female reproductive organs separated in different floral structures on the same plant). As a consequence, the allometric relationships between biomass and seed and pollen numbers are not constant but plastic. Incorporation of these processes into the mechanistic model would not only improve its practical application but potentially contribute to ecological theory seeking to explain the optimal strategy under contrasting environments [42].

In addition to the problem of morphological plasticity, a further challenge to a process based approach to modelling the impact of change on plant populations dynamics is the capacity of biotypes at the limit of the current range to adapt and expand the available niche for colonisation [43]. It is likely that this is the explanation for the poor predictive power of the photothermal time model of *A.artemisiifolia* developed from a single population at a single site, to predict flowering times at the continental scale. Although some progress has been made in terms of predicting the temporal and spatial variability in *A.artemisiifolia* phenology [13], further work is required to understand the importance of adaptation to local conditions of spatially discrete biotypes. It appears that previous assumptions that a relatively simple function of thermal time and daylength is inadequate and a more mechanistic understanding of the interactions of local environment and genetic adaptation is required. This highlights the dangers of basing generic models on data derived from a single population measured at a single site. Incorporating variance in model parameters based on empirical data from populations sampled across an environmental gradient in combination with a sensitivity analysis of the relative importance of processes operating on different stages of the weed life cycle would, therefore, constitute a major advance and improve confidence in model predictions.
